# Supplementary material for: No evidence of positive causal effects of maternal and paternal age at first birth on children’s test scores at age 10 years
Source: Nat Hum Behav. 2025 Feb 27;9(4):731–6. doi: 10.1038/s41562-025-02108-6 (PMC12018448; doi:10.1038/s41562-025-02108-6)
Supplement: Supplementary file 1 — Supplementary Tables 1–3. [file 41562_2025_2108_MOESM1_ESM.pdf]

nature human behaviour

Supplementary information https://doi.org/10.1038/s41562-025-02108-6

No evidence of positive causal effects of maternal and paternal age at first birth on children's test scores at age 10 years

In the format provided by the authors and unedited

TABLE OF CONTENS

Table S1. The associations between maternal and paternal PGIs for age at first birth and age at first birth.

Table S2. The associations between maternal and paternal PGIs for age at first birth and children’s test scores at age 10.

Table S3. Correlation matrix of analysis variables.

Table S1. The associations between maternal and paternal PGIs for age at first birth and age at first birth.

Model number (compare to Table 1)	(1)	(2)	(12)	(13)
		Maternal (N = 15,670)		
Estimate	0.657	0.666	0.335	0.277
95% CI	[0.597, 0.718]	[0.606, 0.727]	[0.261, 0.409]	[0.200 0.353]
BIC	99,156	98,982	91,143	91,112
		Paternal (N = 15,593)		
Estimate	0.433	0.437	0.182	0.171
95% CI	[0.366, 0.500]	[0.370, 0.504]	[0.094, 0.269]	[0.081, 0.260]
BIC	102,871	102,840	95,936	95,945
Model specification:				
PCs 1-10	No	Yes	Yes	Yes
Controls	No	No	Yes	Yes
Parental PGI for education	No	No	No	Yes

Notes: PGIs are standardized, age at first birth is measured in years. The table reports the point estimates and in square brackets the 95% confidence intervals. Controls include grandparental education, partner's PGI for age at first birth, parental education, child PGI for AFB, child's birth cohort (continuous), child's sex, parental PGI age at first sexual intercourse, parental PGI for smoking, parental PGI for contraception use, and parental PGI for ADHD.

Table S2. The associations between maternal and paternal PGIs for age at first birth and children's test scores at age 10.

Model number (compare to Table 1)	(1)	(2)	(12)	(13)
		Maternal (N = 15,670)		
Estimate	0.082	0.082	-0.001	-0.024
95% CI	[0.070, 0.095]	[0.07, 0.095]	[-0.017, 0.016]	[-0.041, -0.008]
BIC	40,352	40,417	37,389	37,262
		Paternal (N = 15,593)		
Estimate	0.084	0.082	-0.002	-0.031
95% CI	[0.071, 0.096]	[0.070, 0.095]	[-0.018, 0.014]	[-0.047, -0.014]
BIC	40,343	40,368	37,015	36,800
Model specification:				
PCs 1-10	No	Yes	Yes	Yes
Controls	No	No	Yes	Yes
Parental PGI for education	No	No	No	Yes

Notes: The outcome is measured in standard deviations, the PGIs are standardized. The table reports the point estimates and in square brackets the 95% confidence intervals. Controls include grandparental education, partner's PGI for age at first birth, parental education, child PGI for AFB, child's birth cohort (continuous), child's sex, parental PGI age at first sexual intercourse, parental PGI for smoking, parental PGI for contraception use, and parental PGI for ADHD.

Table S3. Correlation matrix of analysis variables.

		(1)	(2)	(3)	(4)	(5)	(6)	(7)	(8)	(9)	(10)	(11)	(12)	(13)	(14)	(15)	(16)	(17)	(18)	(19)	(20)	(21)	(22)	(23)	(24)	(25)
	(1)	*1.00*	0.05	0.08	0.11	0.09	0.08	0.06	0.02	0.00	-0.02	0.01	0.04	0.00	0.03	0.00	-0.01	0.02	0.05	0.02	0.01	0.00	0.00	0.01	0.03	0.00
Test score measure	(2)	0.05	*1.00*	0.18	0.26	0.28	0.18	0.12	0.14	0.02	-0.08	0.03	0.30	0.09	0.10	0.02	-0.06	0.03	0.21	0.06	0.10	0.01	-0.06	0.02	0.19	0.07
Grandparental education	(3)	0.08	0.18	*1.00*	0.30	0.26	0.13	0.08	0.09	0.03	-0.06	0.03	0.17	0.05	0.07	0.02	-0.04	0.02	0.14	0.05	0.11	0.02	-0.09	0.03	0.23	0.08
Mother's education	(4)	0.11	0.26	0.30	*1.00*	0.48	0.41	0.29	0.16	0.02	-0.13	0.06	0.26	0.12	0.14	0.02	-0.12	0.04	0.22	0.10	0.18	0.01	-0.15	0.08	0.31	0.16
Father's education	(5)	0.09	0.28	0.26	0.48	*1.00*	0.33	0.23	0.16	0.03	-0.14	0.05	0.28	0.12	0.18	0.02	-0.14	0.06	0.33	0.13	0.15	0.02	-0.11	0.04	0.23	0.11
Mother's age	(6)	0.08	0.18	0.13	0.41	0.33	*1.00*	0.69	0.13	0.02	-0.07	0.05	0.17	0.09	0.12	0.02	-0.07	0.05	0.16	0.08	0.16	0.01	-0.07	0.05	0.19	0.11
Father's age	(7)	0.06	0.12	0.08	0.29	0.23	0.69	*1.00*	0.10	0.01	-0.05	0.04	0.12	0.07	0.10	0.02	-0.04	0.04	0.11	0.07	0.12	0.01	-0.06	0.03	0.14	0.08
Child's PGI for Age at 1st birth	(8)	0.02	0.14	0.09	0.16	0.16	0.13	0.10	*1.00*	0.02	-0.23	0.17	0.40	0.35	0.53	0.02	-0.14	0.10	0.24	0.20	0.53	0.03	-0.13	0.10	0.23	0.19
Child's PGI for ADHD	(9)	0.00	0.02	0.03	0.02	0.03	0.02	0.01	0.02	*1.00*	-0.04	0.00	0.03	0.03	0.03	0.53	-0.05	0.00	0.04	0.03	0.03	0.51	-0.04	0.00	0.02	0.04
Child's PGI for Smoking initiation	(10)	-0.02	-0.08	-0.06	-0.13	-0.14	-0.07	-0.05	-0.23	-0.04	*1.00*	-0.15	-0.24	-0.24	-0.13	-0.03	0.52	-0.07	-0.15	-0.15	-0.13	-0.02	0.54	-0.08	-0.14	-0.14
Child's PGI for Age at 1st contraceptive use	(11)	0.01	0.03	0.03	0.06	0.05	0.05	0.04	0.17	0.00	-0.15	*1.00*	0.12	0.20	0.09	0.02	-0.08	0.50	0.07	0.11	0.10	-0.01	-0.08	0.51	0.07	0.10
Child's PGI for Educational attainment	(12)	0.04	0.30	0.17	0.26	0.28	0.17	0.12	0.40	0.03	-0.24	0.12	*1.00*	0.28	0.24	0.03	-0.15	0.08	0.57	0.17	0.23	0.02	-0.14	0.07	0.56	0.18
Child's PGI for Age at 1st sexual intercourse	(13)	0.00	0.09	0.05	0.12	0.12	0.09	0.07	0.35	0.03	-0.24	0.20	0.28	*1.00*	0.19	0.02	-0.14	0.11	0.17	0.52	0.19	0.03	-0.15	0.10	0.17	0.51
Father's PGI for Age at 1st birth	(14)	0.03	0.10	0.07	0.14	0.18	0.12	0.10	0.53	0.03	-0.13	0.09	0.24	0.19	*1.00*	0.02	-0.22	0.18	0.39	0.36	0.07	0.02	-0.04	0.01	0.09	0.04
Father's PGI for ADHD	(15)	0.00	0.02	0.02	0.02	0.02	0.02	0.02	0.02	0.53	-0.03	0.02	0.03	0.02	0.02	*1.00*	-0.05	0.01	0.04	0.03	0.02	0.02	-0.01	0.00	0.01	0.02

Father's PGI for Smoking initiation	(16)	-0.01	-0.06	-0.04	-0.12	-0.14	-0.07	-0.04	-0.14	-0.05	0.52	-0.08	-0.15	-0.14	-0.22	-0.05	*1.00*	-0.13	-0.22	-0.24	-0.04	-0.02	0.05	-0.01	-0.06	-0.03
Father's PGI for Age at 1st contra-ceptive use	(17)	0.02	0.03	0.02	0.04	0.06	0.05	0.04	0.10	0.00	-0.07	0.50	0.08	0.11	0.18	0.01	-0.13	*1.00*	0.12	0.21	0.02	-0.01	-0.01	0.01	0.02	0.00
Father's PGI for Educa-tional attain-ment	(18)	0.05	0.21	0.14	0.22	0.33	0.16	0.11	0.24	0.04	-0.15	0.07	0.57	0.17	0.39	0.04	-0.22	0.12	*1.00*	0.30	0.08	0.03	-0.06	0.03	0.14	0.07
Father's PGI for Age at 1st sexual intercourse	(19)	0.02	0.06	0.05	0.10	0.13	0.08	0.07	0.20	0.03	-0.15	0.11	0.17	0.52	0.36	0.03	-0.24	0.21	0.30	*1.00*	0.04	0.02	-0.05	0.01	0.06	0.04
Mother's PGI for Age at 1st birth	(20)	0.01	0.10	0.11	0.18	0.15	0.16	0.12	0.53	0.03	-0.13	0.10	0.23	0.19	0.07	0.02	-0.04	0.02	0.08	0.04	*1.00*	0.03	-0.21	0.18	0.39	0.35
Mother's PGI for ADHD	(21)	0.00	0.01	0.02	0.01	0.02	0.01	0.01	0.03	0.51	-0.02	-0.01	0.02	0.03	0.02	0.02	-0.02	-0.01	0.03	0.02	0.03	*1.00*	-0.05	0.00	0.03	0.05
Mother's PGI for Smoking ini-tiation	(22)	0.00	-0.06	-0.09	-0.15	-0.11	-0.07	-0.06	-0.13	-0.04	0.54	-0.08	-0.14	-0.15	-0.04	-0.01	0.05	-0.01	-0.06	-0.05	-0.21	-0.05	*1.00*	-0.14	-0.22	-0.25
Mother's PGI for Age at 1st contra-ceptive use	(23)	0.01	0.02	0.03	0.08	0.04	0.05	0.03	0.10	0.00	-0.08	0.51	0.07	0.10	0.01	0.00	-0.01	0.01	0.03	0.01	0.18	0.00	-0.14	*1.00*	0.12	0.20
Mother's PGI for Edu-cational at-tainment	(24)	0.03	0.19	0.23	0.31	0.23	0.19	0.14	0.23	0.02	-0.14	0.07	0.56	0.17	0.09	0.01	-0.06	0.02	0.14	0.06	0.39	0.03	-0.22	0.12	*1.00*	0.29
Mother's PGI for Age at 1st sexual intercourse	(25)	0.00	0.07	0.08	0.16	0.11	0.11	0.08	0.19	0.04	-0.14	0.10	0.18	0.51	0.04	0.02	-0.03	0.00	0.07	0.04	0.35	0.05	-0.25	0.20	0.29	*1.00*
