# Supplementary material for: No evidence of positive causal effects of maternal and paternal age at first birth on children’s test scores at age 10 years
Source: Nat Hum Behav. 2025 Feb 27;9(4):731–6. doi: 10.1038/s41562-025-02108-6 (PMC12018448; doi:10.1038/s41562-025-02108-6)
Supplement: Supplementary file 2 — Reporting Summary [file 41562_2025_2108_MOESM2_ESM.pdf]

nature portfolio

Corresponding author(s): Michael Grätz

Last updated by author(s): 2024/11/17

Reporting Summary

Nature Portfolio wishes to improve the reproducibility of the work that we publish. This form provides structure for consistency and transparency in reporting. For further information on Nature Portfolio policies, see our Editorial Policies and the Editorial Policy Checklist.

Statistics

For all statistical analyses, confirm that the following items are present in the figure legend, table legend, main text, or Methods section.

n/a	Confirmed	
☐	☒	The exact sample size (*n*) for each experimental group/condition, given as a discrete number and unit of measurement
☐	☒	A statement on whether measurements were taken from distinct samples or whether the same sample was measured repeatedly
☐	☒	The statistical test(s) used AND whether they are one- or two-sided<br>*Only common tests should be described solely by name; describe more complex techniques in the Methods section.*
☐	☒	A description of all covariates tested
☐	☒	A description of any assumptions or corrections, such as tests of normality and adjustment for multiple comparisons
☐	☒	A full description of the statistical parameters including central tendency (e.g. means) or other basic estimates (e.g. regression coefficient) AND variation (e.g. standard deviation) or associated estimates of uncertainty (e.g. confidence intervals)
☐	☒	For null hypothesis testing, the test statistic (e.g. *F*, *t*, *r*) with confidence intervals, effect sizes, degrees of freedom and *P* value noted<br>*Give P values as exact values whenever suitable.*
☒	☐	For Bayesian analysis, information on the choice of priors and Markov chain Monte Carlo settings
☒	☐	For hierarchical and complex designs, identification of the appropriate level for tests and full reporting of outcomes
☒	☐	Estimates of effect sizes (e.g. Cohen's *d*, Pearson's *r*), indicating how they were calculated

Our web collection on statistics for biologists contains articles on many of the points above.

Software and code

Policy information about availability of computer code

Data collection	NA
Data analysis	Data analysis were conducted using R. The R code to replicate all analyses is available at https://github.com/torkildl/parental-age.

For manuscripts utilizing custom algorithms or software that are central to the research but not yet described in published literature, software must be made available to editors and reviewers. We strongly encourage code deposition in a community repository (e.g. GitHub). See the Nature Portfolio guidelines for submitting code & software for further information.

Data

Policy information about availability of data

All manuscripts must include a data availability statement. This statement should provide the following information, where applicable:

- Accession codes, unique identifiers, or web links for publicly available datasets
- A description of any restrictions on data availability
- For clinical datasets or third party data, please ensure that the statement adheres to our policy

The MoBa data is available under restricted access due to data protection laws, and access can be obtained by application to MoBa and a Regional Committee for Medical and Health Research Ethics in Norway. If you wish to apply for access, please visit the following website for more details: https://www.fhi.no/en/ch/studies/moba/for-forskere-artikler/research-and-data-access/.

Research involving human participants, their data, or biological material

Policy information about studies with human participants or human data. See also policy information about sex, gender (identity/presentation), and sexual orientation and race, ethnicity and racism.

Reporting on sex and gender	Mothers and Fathers are analysed seperately in our study. Sex has been self-reported.
Reporting on race, ethnicity, or other socially relevant groupings	NA
Population characteristics	Our regression models control of age of the participants, grandparental education, population stratification using genetic principal components, polygenic scores for age at first birth, age at first sexual intercourse, age at smoking initiation, age at first use of oral contraceptives, attention deficit hyperactivity disorder, educational attainment, for age and sex of the children.
Recruitment	The data consists of a population based cohort study conducted by the Norwegian Institute of Public Health. Pregnant women were recruited in Norway from 1999 to 2008. Women consented to participate in 41% of cases. All participants consented. No compensation was paid.
Ethics oversight	Regional Committee for Medical and Health Research Ethics in Norway

Note that full information on the approval of the study protocol must also be provided in the manuscript.

Field-specific reporting

Please select the one below that is the best fit for your research. If you are not sure, read the appropriate sections before making your selection.

☐ Life sciences ☒ Behavioural & social sciences ☐ Ecological, evolutionary & environmental sciences

For a reference copy of the document with all sections, see nature.com/documents/nr-reporting-summary-flat.pdf

Behavioural & social sciences study design

All studies must disclose on these points even when the disclosure is negative.

Study description	We conduct a quantitative Mendelian Randomization analyses using IV regression models.
Research sample	We use existing data from the Norwegian Mother, Father, Child Cohort Study, a population based sample of pregnant women in Norway conducted from 1999 to 2008. This study has both parents and the children resulting from the pregnancy genotyped which allowed us for some statistical modeling that is quite unique to our study.
Sampling strategy	All pregnant women attending a routine ultrasound examination were invited by the data collectors. We did not collect data ourselves.
Data collection	Blood samples were collected from fathers and mothers and questionnaires were sent to both parents. Records were linked to existing registry data.
Timing	10/99 - 07/08
Data exclusions	Of all MoBa children (114500), we used information on those children (17523) who themselves were genotyped, had genotyped parents and were the first-born child of both parents.
Non-participation	The response rate was 41%
Randomization	There was no randomization, it's a population-based data set. We used multivariate statistical models for causal inference.

Reporting for specific materials, systems and methods

We require information from authors about some types of materials, experimental systems and methods used in many studies. Here, indicate whether each material, system or method listed is relevant to your study. If you are not sure if a list item applies to your research, read the appropriate section before selecting a response.

Materials & experimental systems

n/a	Involved in the study
☒	☐ Antibodies
☒	☐ Eukaryotic cell lines
☒	☐ Palaeontology and archaeology
☒	☐ Animals and other organisms
☒	☐ Clinical data
☒	☐ Dual use research of concern
☒	☐ Plants

Methods

n/a	Involved in the study
☒	☐ ChIP-seq
☒	☐ Flow cytometry
☒	☐ MRI-based neuroimaging
